# Psychological interventions for patients with delirium in intensive care: A scoping review protocol

**DOI:** 10.1371/journal.pone.0315832

**Published:** 2024-12-20

**Authors:** Madiha Shaikh, Dorothy M. Wade, Lara King, Liam Mackay, Isabelle Symes, Anam Syeda, Anna Greenburgh

**Affiliations:** 1 Clinical Health Psychology, North East London Foundation NHS Trust, United Kingdom; 2 Research Department of Clinical, Educational and Health, University College London, United Kingdom; 3 Royal Free London NHS Foundation Trust, United Kingdom; 4 Department of Behavioural Science and Health, University College London, United Kingdom; 5 Division of Psychology and Language Sciences, University College London, United Kingdom; 6 Division of Psychiatry, University College London (UCL), United Kingdom; 7 Digital Psychological Service, NHS Lanarkshire, United Kingdom; 8 Institute of Epidemiology & Health Care, University College London, United Kingdom; 9 ESRC Centre for Society and Mental Health, United Kingdom; 10 Department of Health Service and Population Research, King’s College London, United Kingdom; University of Toronto, CANADA

## Abstract

The objective of this scoping review is to investigate psychological interventions developed, evaluated, and considered for patients with delirium in intensive care units (ICU). Data will be extracted from sources of evidence that address interventions for delirium-related distress and/or cognitive impairments in the adult ICU population, suitable for delivery by or under the supervision of a psychological practitioner. ICU delirium is a common and impactful condition that adversely affects patient outcomes, including prolonged hospitalisation and deteriorating mental health. Despite its significance, it remains poorly understood. Addressing the psychological impact of delirium is crucial for improving both short- and long-term psychological outcomes in ICU patients. However, current non-pharmacological interventions often fail to consider this issue. The inclusion criteria encompass psychological interventions for critically ill adults that directly impact their thoughts, feelings, behaviour and/or cognition. Additionally, interventions involving relatives and multi-component non-pharmacological approaches will be considered. The databases Medline (Ovid), PsycINFO (Ovid), Embase (Ovid), and CINAHL Plus will be searched, covering literature from 1990 to the present. We chose 1990 as the earliest time point for searches because psychological input in ICUs began globally around 2000, with notable expansion in the past decade. Reference lists from identified articles will be hand-searched and PsycEXTRA (Ovid) and WorldCat.org will be searched for grey literature. Relevant information will be extracted and reported using a PRISMA flow diagram, characteristics and frequency table as well as narrative descriptions. This review aims to collate evidence to guide the development and evaluation of new psychological interventions to address delirium in the ICU.

## Introduction

Delirium is an acute confusional state characterised by a fluctuating disturbance of attention, consciousness, cognition, and reduced orientation to the environment [1]. Delirium is common in intensive care units (ICUs), with an incidence ranging from 31% to 80% [2, 3]. It is typically diagnosed using clinical assessment tools, such as the Confusion Assessment Method for the ICU (CAM-ICU) or the Intensive Care Delirium Screening Checklist (ICDSC), which assess changes in attention, awareness, and cognitive function. Adverse outcomes include prolonged mechanical ventilation and hospitalization, worse mental health outcomes, cognitive impairment, and increased mortality [4, 5]. Additionally, delirium is associated with increased anxiety and stress for patients, families and ICU staff [6, 7]. These adverse outcomes contribute to higher healthcare costs.

A rigorous consultation exercise supported by the National Institute for Health Research (NIHR) identified this issue as particularly important for patients, families, and staff, who rated the detection and management of ICU delirium as one of three top priorities for ICU research [8]. In a large international consensus study, ICU patients and families selected emotional distress as the single most important of 100 possible outcomes of delirium to be measured in research [9]. Yet little work has been done in alleviating the distress experienced by delirious ICU patients.

Multiple factors contribute to delirium, including medication, other aspects of illness and treatment, and environmental and psychological stressors [2]. Pharmacological treatment of delirium with benzodiazepines or antipsychotics is common in ICUs but evidence for their efficacy is weak [10].

Non-pharmacological interventions for delirium have been evaluated in different hospital settings. The Hospital Elder Life Program (HELP), including cognitive impairment management, sleep hygiene, and early mobility, has shown positive effects in reducing delirium incidence in hospitalised elderly patients [11]. In the ICU setting, the ABCDEF (Assess, prevent, and manage pain; Both spontaneous awakening trials and breathing trials; Choice of analgesia and sedation; Delirium: assess, prevent, and manage; Early mobility and exercise; Family engagement and empowerment) bundle improved symptoms of delirium and other outcomes in a cohort of more than 15,000 patients [12]. The psychological impact of delirium, including emotional distress and cognitive impairments, is inadequately addressed in most non-pharmacological bundles. Research indicates that patients with ICU delirium often experience emotions such as anger, fear, and shame, along with cognitive difficulties with memory, thinking, orientation, and perception [13]. Many ICU patients with delirium experience hallucinations and/or delusions, and literature suggests that the memory of these experiences can be deeply distressing [14]. Hallucinations are sensory perceptions that occur without external stimuli, while delusions are strongly held beliefs despite evidence to the contrary. Many ICU patient experience long-term mental health challenges such as depression, post-traumatic stress disorder, and cognitive impairments [15]. Our hypothesis is that addressing the psychological components of ICU delirium would help improve short and long-term psychological outcomes of patients. Qualified psychologists have a range of evidence-based methods for managing emotional distress and cognitive impairments that could be applied to this issue. However, there is currently a paucity of evidence available to support our hypothesis.

Scoping reviews are exploratory projects that descriptively map, report, and discuss the extent of the literature available on a topic, clarify key concepts, theories, sources of evidence, and knowledge gaps [16]. This approach to evidence synthesis offers a valuable overview of research questions reported in the literature that have received scant attention thus far. It has gained rapid international popularity owing to its effectiveness in navigating complex and heterogeneous literature [17]. There are multiple purposes for conducting a scoping review [18], and some that informed this paper include:

- To examine the breadth and depth of literature on a certain topic or field: A scoping review enables a comprehensive exploration of the range of non-pharmacological interventions, including those that may not be covered in a more narrowly focused systematic review.
- To identify or analyse knowledge gaps: This approach is well-suited to identifying research gaps, particularly important given the likely limited literature in this field, especially within intensive care settings.
- To clarify key characteristics and definitions related to a concept. Scoping reviews help clarify the diverse characteristics and definitions of interventions, offering a more nuanced understanding of how they are applied in practice.
- Inclusion of Grey Literature: Scoping reviews include grey literature, such as reports and conference papers, which is crucial for gaining insights in a field where peer-reviewed studies may be sparse.
- Flexibility in Study Inclusion: The flexibility of a scoping review allows for the inclusion of various study types, including qualitative research on the experiences of delivering interventions, providing a richer understanding of real-world applications.

Given the emerging and under-explored nature of non-pharmacological interventions for delirium in intensive care, a scoping review is the most appropriate method to comprehensively map the existing evidence, identify gaps, and inform future research directions.

### Review aim

This scoping review aims to explore the landscape of psychological interventions that have been developed, evaluated, or considered for managing delirium in adult ICU patients. The evidence gathered from this review will play a crucial role in informing the development and evaluation of novel psychological interventions tailored specifically for ICU delirium.

## Methodology

### Identifying the research question

The proposed scoping review will follow the JBI methodology for scoping reviews [18, 19], adapted from the foundational Arksey and O’Malley [17] scoping review methodology. This review will also utilise the PRISMA extension for scoping reviews (PRISMA-ScR) checklist [20]. The quality of relevant literature will not be appraised. Nine steps will be followed: developing and aligning the inclusion criteria with the objective/s and question/s, describing the planned approach to evidence searching, selection, data extraction, and presentation of the evidence, searching for the evidence, selecting the evidence, extracting the evidence, analysis of the evidence, presentation of the results, summarising the evidence in relation to the purpose of the review, making conclusions and noting any implications of the findings.

The main research question of this scoping review is “What psychological interventions are available to address emotional distress and cognitive impairments associated with delirium in intensive care settings?”

#### Inclusion criteria

The review will consider a variety of sources of evidence for inclusion, including primary empirical research studies, systematic reviews, meta-analyses, letters, guidelines, and conference papers. These should address:

- Participants admitted into critical care (CC), a high dependency unit (HDU) or intensive care unit (ICU), aged 18 years and older, and with a diagnosis of delirium.
- Psychological interventions that directly impact thoughts, feelings (emotions or physical sensations), behaviour and/or cognition.
- Interventions should be specifically suitable for delivery by a psychology professional, or by other care professionals with training and supervision by psychologists.
- Interventions may involve work with relatives/valued others that would have an indirect impact on the thoughts, feelings, behaviour and/or cognition of the person experiencing delirium.
- Multi-component non-pharmacological interventions that include significant and well-described psychological components (even if other types of clinical component are included).

#### Exclusion criteria

We will exclude literature that addresses:

- Interventions including only physical or environmental components
- Interventions that are not suitable for delivery or supervision by a psychologist
- Interventions for child and adolescent samples (i.e., under 18)
- Interventions that do not directly or indirectly influence thoughts, feelings, behaviour and/or cognition.

### Identify the relevant literature (Search strategy)

The following electronic databases will be searched for published literature related to this research field, namely Medline (Ovid), PsycINFO (Ovid), Embase (Ovid), and CINAHL Plus. To locate grey literature, PsycEXTRA (Ovid) and WorldCat.org will be searched to ensure hard-to-reach papers are considered. Reference lists of identified studies will be hand-searched to find further potential sources. We used the PICO framework to develop search terms with the assistance of a librarian at the University College London to ensure the search strategy was suitable. The search terms include five aspects: i) clinical condition ii) setting iii) type of intervention iv) intervention specification v) age (see Appendix A in S1 File). For clinical conditions, terms such as delirium, agitation, and confusion will be included. For settings, terms such as intensive care, critical care, or ICU will be included. For the type of intervention, terms such as non-pharmacological, psychological, behavioural, cognitive, and emotional will be included. For intervention specification, terms such as intervention, treatment, therapy, prevention, bundle, care, and support will be included. For age, studies with headings that include children will be removed. To ensure we capture all relevant studies, we will include MeSH terms/subject headings and will use truncation where terms may have multiple spellings. After the initial search, the search will be repeated immediately prior to the write-up to ensure the inclusion of all relevant data within the time frame.

### Study selection

Three reviewers will independently conduct each stage of the review process in duplicate using EndNote. Title and abstract screening will be performed, and any uncertainties regarding the inclusion of sources will be resolved through discussion and consensus among the reviewers and if required senior authors. During full-text screening, the reviewers will apply additional inclusion and exclusion criteria to ensure selected literature aligns with the research question. Additional exclusions may involve papers lacking a psychological component, unrelated to delirium, not referencing non-pharmacological techniques, or inaccessible. The reasons for retaining eligible sources will be documented. The selection process will be captured in a PRISMA flow diagram.

### Data extraction

Relevant information will be recorded electronically from each source in a data extraction form, which will include the name of author(s), year of publication, country of origin, type and setting, and any key findings that relate to the research question. Further information recorded for empirical studies includes population, age, and type of psychological intervention (including duration of intervention, and outcome measures where applicable).

The process of data extraction will involve three reviewers to reduce the likelihood of bias and errors. The data extraction method will be planned and familiarised with at the training stage, with a pilot conducted across reviewers to ensure that the method is effective and feasible.

### Collating, summarising, and reporting the results

Our results section will aim to capture the nature and extent of literature that covers psychological interventions for ICU delirium. All data will be presented as originally reported in the papers and key findings of each paper will be summarised. As is standard practice for scoping reviews, we will not assess the quality of evidence and/or synthesise the data within our data.

The extracted data will be presented in both tabular (see Appendix B in S1 File for draft tables) and descriptive forms. In the Table 1 in S1 File, we will present the characteristics, design (where relevant), and findings of each included paper. This will include author/date, paper type, population (N), setting/country, type of psychological intervention, single/bundle intervention, outcome indicators, and findings. Additionally, we will provide Table 2 in S1 File, a frequency table to map the extent of the available literature, including the frequencies of paper type, setting/country, psychological intervention type, target of intervention, single or bundle intervention, outcome indicators, and findings. A narrative summary of the scoping review’s findings will also be provided to clarify concepts, draw conclusions, consider implications for both research and clinical practice, and identify research gaps in the literature.

## Supporting information

S1 File (DOCX)
