# Supplementary material for: Psychological interventions for patients with delirium in intensive care: A scoping review protocol
Source: PLoS One. 2024 Dec 20;19(12):e0315832. doi: 10.1371/journal.pone.0315832 (PMC11661615; doi:10.1371/journal.pone.0315832)
Supplement: S1 File — (DOCX) [file pone.0315832.s001.docx]

**Appendix A**

Search strategy.

| Database | Limits | Search Terms | Records Identified |
| --- | --- | --- | --- |
| Medline (Ovid) | 1990-present  Age: 18 years and older | deliri* OR agitat* OR confus*  AND  intensive care OR critical care OR ICU  AND  non-pharm* OR nonpharm* OR psych* OR behavio?r* OR cogniti* OR emotion*  AND  interven* OR treat* OR therap* OR prevent* OR bundle OR care OR support |  |

**Detailed sample search for Medline:**

1. **Search Terms for delirium, agitation, or confusion:**
   - **(Deliri* or agitat* or confus*) in title/abstract: .tw.**
2. **MeSH Term for delirium:**
   - **Delirium/**
3. **Combine the above searches for delirium-related terms:**
   - **1 or 2**
4. **Search Terms for ICU-related terms:**
   - **(Intensive care or critical care or ICU) in title/abstract: .tw.**
5. **MeSH Terms for critical illness or ICU:**
   - **Critical Illness/, Critical Care/, Intensive Care Units/, Intensive Care/**
6. **Combine the above ICU-related searches:**
   - **4 or 5**
7. **Search Terms for non-pharmacological, psychological, or behavioural interventions:**
   - **(Non-pharm* or nonpharm* or Psych* or behavio?r* or cogniti* or emotion*) in title/abstract: .tw.**
8. **Search Terms for intervention-related terms:**
   - **(interven* or treat* or therap* or prevent* or bundle or care or support*) in title/abstract: .tw.**
9. **Combine the results of delirium, ICU, and intervention terms:**
   - **3 and 6 and 7 and 8**
10. **MeSH Term to exclude adult-related studies and focus on children:**
    - **exp child/ not exp adult/**
11. **MeSH Terms for neonatal and pediatric intensive care:**
    - **Intensive Care, Neonatal/, Intensive Care Units, Pediatric/**
12. **Search Terms for child-related terms (excluding adult):**
    - **((child* or neonat* or infant* or p?ediatric*) not adult*) in title/abstract: .tw.**
13. **Combine child-related searches:**
    - **10 or 11 or 12**
14. **Exclude child-related studies from the combined results:**
    - **9 not 13**

**Appendix B**

**Table 1**

Draft table of characteristics, designs, and findings of papers.

| Author/Date | Study design | Population (N) | Setting/Country | Diagnosis of delirium | Type of psychological intervention | Single/bundle intervention | Outcome indicators/  measures | Findings |
| --- | --- | --- | --- | --- | --- | --- | --- | --- |
| … | e.g. primary empirical research studies | e.g. patients aged 18 and older. | e.g. intensive care unit (ICU)  /Australia | e.g. CAM ICU | e.g. cognitive rehabilitation | e.g. single | e.g. delirium-related distress | … |
| … | e.g. systematic review | e.g. older adults | e.g. high dependency unit (HDU)/France |  | e.g. distress management | e.g. bundle | e.g. feasibility | … |

**Table 2**

Draft frequency table

| Domain | Sub-categories | Frequency (N/%) |
| --- | --- | --- |
| Publications | Total |  |
|  |  |  |
| Paper type | Primary empirical research studies |  |
|  | Systematic reviews |  |
|  | Meta-analyses |  |
|  | Letters |  |
|  | Guidelines |  |
|  | Conference papers |  |
| Setting/country | Critical care (CC)  High dependency unit (HDU)  Intensive care unit (ICU)  e.g. *  Australia |  |
| Diagnosis of delirium | e.g. CAM ICU |  |
|  |  |  |
| Type of psychological intervention | e.g.*  Cognitive rehabilitation exercises  Family education |  |
|  |  |  |
| Recipient of intervention | Patient-only  Patient and family  Family-only |  |
| Single/bundle intervention | Single  Bundle |  |
| Outcome indicators/measures | e.g. *  Delirium-related distress  Severity of delirium  Feasibility of implementing intervention |  |

Sub-categories will be specified after review is conducted
